# Efficacy and Safety of Gefitinib in Patients with Advanced Head and Neck Squamous Cell Carcinoma: A Meta-Analysis of Randomized Controlled Trials

**DOI:** 10.1155/2019/6273438

**Published:** 2019-05-23

**Authors:** Xiaoxia Tang, Juan He, Bo Li, Yi Zheng, Kejia Li, Shun Zou, Long Chen

**Affiliations:** ^1^Department of Pharmacy, The Second Affiliated Hospital of Kunming Medical University, Kunming, Yunnan, China; ^2^Department of Pharmacy, The 920th Hospital of PLA, Kunming, Yunnan, China; ^3^College of Pharmaceutical Sciences, Kunming Medical University, Kunming, China; ^4^Advanced Analytics Institute, University of Technology Sydney, Australia; ^5^Department of PET/CT Center, Yunnan Cancer Hospital, The Third Affiliated Hospital of Kunming Medical University, Yunnan, China

## Abstract

**Background:**

Trials on assessing the benefits of EGFR inhibitors in head and neck squamous cell carcinoma (HNSCC) patients have gradually been published. Nevertheless, the benefits of gefitinib in advanced HNSCC are still unknown.

**Methods:**

The Cochrane library, PubMed, and EMBASE databases were systematically searched from the inception dates to 17 July 2017, 18 July 2017, and 19 July 2017, respectively. The keywords “head and neck” and gefitinib were used to retrieve in articles and abstracts. An additional search for recently published randomized trials was performed from July 17, 2017, to April 18, 2018. Then we assessed the risk of bias of the included studies based on the Cochrane “Risk of Bias” tool. Quantitative analysis was carried out to evaluate the overall survival (OS), progression free survival (PFS), overall response rate (ORR), and grade 3-4 adverse effects by Review Manager 5.0.2 and the quality-of-life was analyzed in the included studies.

**Results:**

Seven randomized controlled trials and a total number of 1287 patients were involved. There were no significant differences in OS, PFS, or ORR between gefitinib and no gefitinib group (HR 1.07, 95% CI 0.93 to 1.22, and P=0.35; HR 0.84, 95% CI 0.69 to 1.04, and P=0.11; RR 1.04, 95% CI 0.90 to 1.20, and P =0.60, respectively). However, gefitinib alone was equivalent to chemotherapeutics (i.e., methotrexate; methotrexate + fluorouracil) in ORR in patients with recurrent HNSCC, and a trend of improvement in QOL in gefitinib group was showed. Toxicities revealed no differences except for diarrhea and skin toxicity (p=0.0003; p=0.03, respectively).

**Conclusion:**

For patients with advanced HNSCC, gefitinib cannot prolong the OS and PFS or improve ORR, and odds of skin toxicity and diarrhea increased. However, gefitinib alone is equivalent to methotrexate or methotrexate + fluorouracil and tends to improve QOL for recurrent patients.

## 1. Introduction

Head and neck cancer is one of the leading causes of cancer deaths in the world. Squamous cell carcinoma and its variants account for more than 90% of these tumors [1], involving cancers of lip, oral cavity, pharynx, larynx, and paranasal sinuses. It is estimated that about 300,400 new cases and 145,400 deaths from lip cancer and oral cavity cancer occurred in 2012 worldwide [2], and approximately 63,030 new oral cavity, pharynx, and larynx cancer cases and 13,360 deaths occurred in the United States during 2017 [3]. For decades, oral cavity cancer incidence rates have decreased in Asia, Northern America, and Australia. However, with the ongoing tobacco epidemic, the rates increased in several countries of Eastern and Northern Europe and among females in Southern and Western Europe [2]. Approximately two-thirds of head and neck squamous cell carcinomas (HNSCCs) present in advanced stage, including local stage (stage III/IVa/IVb) and metastatic stage (stage IVc) [4]. Surgery cannot achieve satisfactory results due to limitations of gender, age, and health status. A concomitant or alternating systemic therapy is used for treatment, which was limited by the high incidence of resistance, lack of specificity, and unacceptable adverse effects of radiotherapy and chemotherapy. Over the past years, the incidence of local recurrence and distant metastasis has increased, and the survival benefit of HNSCC has reached the bottleneck stage. Drugs with high efficiency, high selectivity, low toxicity, and reverse resistance of radiotherapy and chemotherapy are badly needed. EGFR is a member of a family of receptor tyrosine kinases, which was expressed in the majority of epithelial malignancies including breast, glioblastoma, and squamous cell carcinoma [5]. According to Ang et al. [6], 95% of the HNSCCs had detectable EGFR expression, and more than 90% were overexpressed. Compared to lower EGFR-expressing HNSCCs patients, these with higher EGFR-expressing had significantly poorer overall survival (OS) and disease-free survival (DFS) and lower local-regional control (LRC). There are two major classes of EGFR-targeted drugs: the extracellular domain of the receptor monoclonal antibody (MAb) and the acting on the receptor intracellular region of small molecule tyrosine kinase inhibitors (TKIs). It is now well established from a variety of studies [7, 8] that, for patients with HNSCCs, cetuximab confers a statistically significant improvement in OS, which was approved by the U.S. Food and Drug Administration (FDA) in 2006 to treat HNSCC [9]. One of the other types of EGFR-blocking drug known as TKI, gefitinib is effective in the treatment of lung cancer with EGFR mutations; however, its benefits in HNSCCs are unknown. Trials on assessing the benefits of gefitinib in HNSCCs patients have recently been published. Therefore, a meta-analysis was performed to clarify the effectiveness and safety of gefitinib for advanced head and neck squamous cell carcinoma. To be specific, since the prognosis of metastatic HNSCC is very similar to that of recurrent HNSCC, advanced HNSCC is often divided into locoregionally advanced (LA) stage and recurrent/metastatic (RM) stage [4]. Therefore, the “advanced disease” of HNSCC in the current study includes both LA stage and RM stage.

## 2. Methods

### 2.1. Search Methods

We searched the Cochrane Central Register of Controlled Trials (CENTRAL), the Cochrane Library on 17 July 2017; PubMed databases on 18 July 2017; Embase databases on 19 July 2017. The keywords “head and neck” and gefitinib were used to retrieve in articles and abstracts. In addition, we searched the reference lists of included trials and contacted experts in the field. An additional search was performed from July 17, 2017, to April 18, 2018, to identify recently published RCTs of those meeting the inclusion criteria, using the databases and keywords described above.

### 2.2. Inclusion Criteria

Randomized controlled trials were included based on the following inclusion criteria: (1) written in English, (2) involving patients diagnosed with biopsy-proven HNSCC, and (3) the studies that reported hazard ratios (HRs) for OS or progression-free survival (PFS) or both, response rate or grade 3-4 adverse drug reactions. At the same time, studies were excluded based on the following criteria: (1) Trials for nasopharynx cancer or esophageal cancer were excluded because these cancers differ from other head and neck cancers in etiology, epidemiology, histological type, and therapeutic schedules [10]. (2) Studies whose full text cannot be obtained were excluded as well.

### 2.3. Risk-of-Bias Assessments

The methodological quality for all included RCTs was evaluated according to Cochrane risk-of-bias criteria, including the randomization sequence generation, allocation concealment, blinding, incomplete outcome data, selective reporting, and other biases. And each quality item was graded as low risk, high risk, or unclear risk.

### 2.4. Data Extraction

The following information as well as the experiment data from all obtained studies were extracted: the author's name, publication year, gender, country, number of patients, mean age, therapy regimens, the hazard ratio (HR) and 95% CI of either overall survival (OS) and/or PFS, overall response rate(ORR), quality-of-life (QOL), and grade 3-4 adverse effect.

### 2.5. Statistical Analysis

We performed meta-analysis to calculate the overall HR and its 95% CI for OS and PFS. Relative risk (RR) and its 95% CI were calculated for ORR and grade 3-4 toxicities. A statistical test with p<0.05 is considered a significant outcome, while a* P* value >0.05 indicates no significant difference between the two comparison arms. Standard Q test and I^2^ statistic were used to estimate statistical heterogeneity among trials. Heterogeneity exists in the pooled HRs or RRs when* P* values<0.10 or I^2^>50% [11]. When homogeneity is deemed invalid (p<0.10, I^2^>50%), a random-effect model is applied for secondary analysis; otherwise, a fixed-effect model was used. The meta-analyses were performed using Review Manager Version 5.0.2 (the Cochrane collaboration). Potential publication bias was evaluated by Begg's funnel plot and Egger's tests, which were performed using STATA version 12.0 (STATA Corp, College Station, TX). And* P* < 0.05 represents significant publication bias.

## 3. Results

### 3.1. Eligible Studies and Characteristics

**Figure 1:**
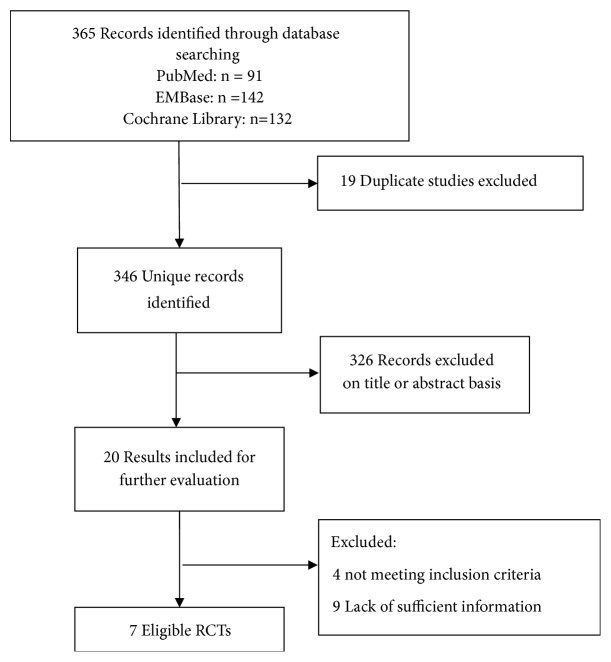
Literature search and screening process.

First, we identified 365 potentially eligible records in the initial search and obtained 346 records after deduplication. Then we selected 20 reports from them for further evaluation after screening their titles and abstracts. Finally, we obtained seven records [12–18] which meet the inclusion criteria for our study (Figure 1). The 7 selected papers were published between 2009 and 2016 and involved a total number of 1287 patients.  Table 1 reports the characteristics of the included RCTs.

### 3.2. Risk of Bias in Included Studies

**Figure 2:**
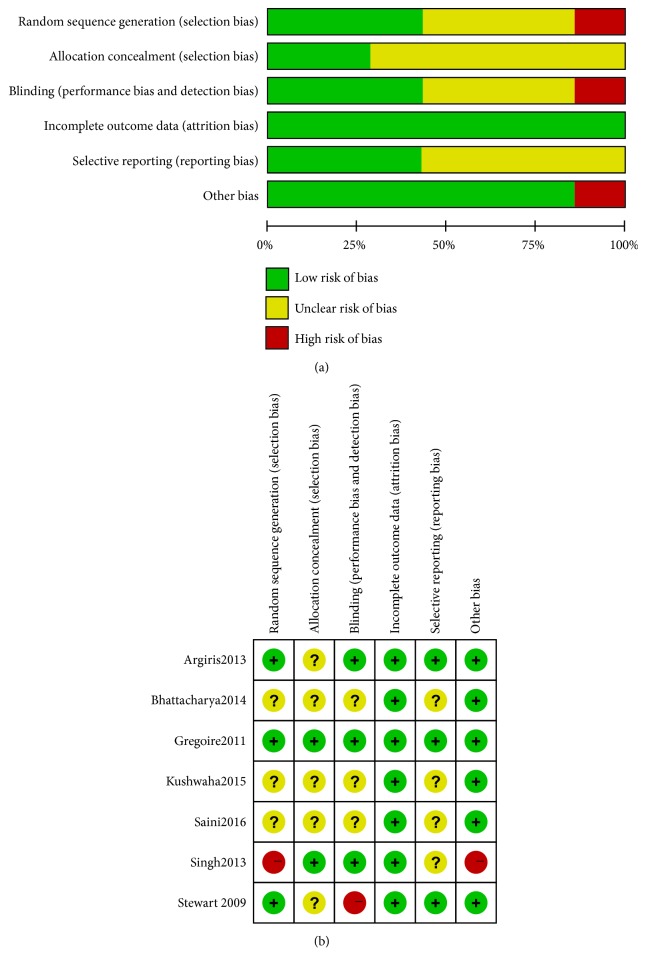
Risk of bias. (a) Risk of bias graph based on the Cochrane “Risk of bias” tool. (b) Risk of bias summary based on the Cochrane “Risk of bias” tool.

The included studies for risk of bias were assessed based on the Cochrane “Risk of Bias” tool. Results of the overall “Risk of Bias” assessment are displayed in Figure 2(a) and a summary of the risk of bias for each included trial was displayed in Figure 2(b). All included studies stated that they were “randomized.” Three trials [12, 13, 15] described an adequate random sequence generation process only, and one trial [16] performed imbalanced randomization. Allocation was adequately concealed in two studies [12, 16], and the remaining five studies did not report the method of allocation concealment. Three trials [12, 15, 16] were double blind, while one trial [13] allocation was open-label; the other trials were unclear. Four articles [12, 13, 16, 17] have reported complete outcome data, and the other three articles [14, 15, 18] explicitly provided the number and reasons for withdrawal or loss to follow-up. In three of the included papers [12, 13, 15], the protocol is available. Others are not, which were judged the risk of reporting bias as unclear. One study [16] with imbalanced baseline characteristics was performed at their institute, so it was left as high risk of bias for this domain.

### 3.3. Overall Survival (OS)

**Figure 3:**
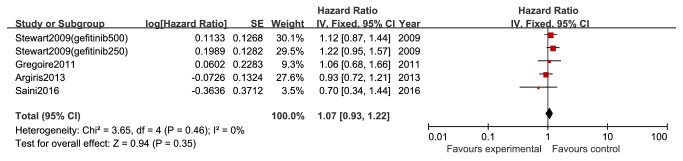
OS between gefitinib and no gefitinib group in patients with advanced head and neck cancer (HR 1.07; Z=0.94; P=0.35).

Four records were included in this meta-analysis, which reported the hazard ratios (HR) and their 95% CIs for OS. There were no significant differences in OS between gefitinib and no gefitinib group (HR 1.07, 95% CI 0.93 to 1.22, and P=0.35; Figure 3).

### 3.4. Progression-Free Survival (PFS)

**Figure 4:**
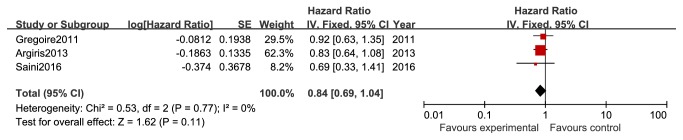
PFS between gefitinib and no gefitinib group in patients with advanced head and neck cancer (HR 0.84; Z=1.62; P=0.11).

On the basis of the three studies that reported the hazard ratios (HR) and their 95% CIs for PFS, no significant differences were found between patients with gefitinib and no gefitinib (HR 0.84, 95% CI 0.69 to 1.04, and P=0.11; Figure 4).

### 3.5. Overall Response Rate (ORR)

**Figure 5:**
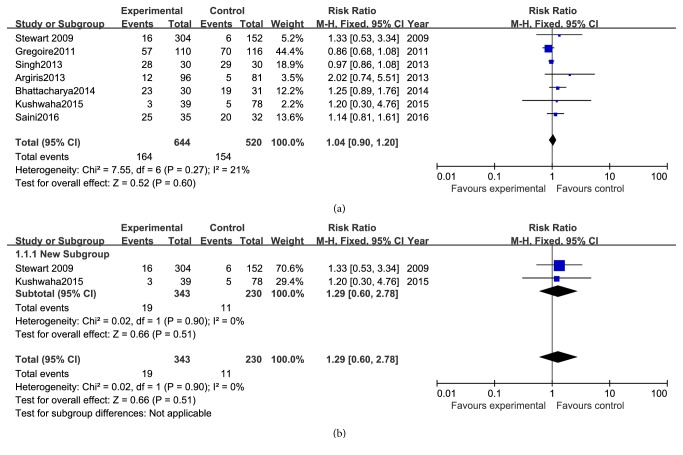
ORR between gefitinib and no gefitinib group in different patients. (a) ORR between gefitinib and no gefitinib group in patients with advanced head and neck cancer (RR: 1.04; Z=0.52; P = 0.60). (b) ORR between gefitinib and no gefitinib group in patients with recurrent head and neck cancer (RR: 1.29; Z=0.66; P = 0.51).

All the seven reports had data on ORR, and the RR analysis revealed no advantage for gefitinib-based treatment (Figure 5(a)) in patients with advanced head and neck cancer (RR 1.04, 95% CI 0.90 to 1.20, and P = 0.60).

Moreover, we conduct subgroup analyses of the effectiveness of gefitinib in patients with recurrent squamous cell carcinoma of the head and neck. The results from two studies indicated that they are equivalent in ORR (RR 1.29, 95% CI 0.60 to 2.78, and P = 0.51; Figure 5(b)) between gefitinib alone and chemotherapeutics, such as methotrexate and methotrexate + fluorouracil.

### 3.6. Quality-of-Life (QOL)

QOL was analyzed in three of the included studies, which involved recurrent HNSCC patients. Kushwaha et al. [14] measured QOL with the instrument FHNSI-10 only. Stewart et al. [13] assessed QOL combining FHNSI-10 with the FACTH&N questionnaires. Bhattacharya et al. evaluated the QOL using the EORTC QLQ-C30 (version 3.0) and the QLQ-H and N35 instruments [18]. Due to the different evaluation methods, data cannot be derived. Therefore, descriptive analysis was carried out. Stewart et al. [13] demonstrated a trend of improvement in QOL measures in patients randomly assigned to gefitinib, although these improvements were not statistically significant. The results of Bhattacharya suggested that gefitinib group has a better QOL than the control group in locally advanced HNSCC [18], but they did not conduct statistical analysis of the results. Kushwaha's result that demonstrated significant improvements in QOL with manageable toxicities was observed in gefitinib arm (P<0.001) [14].

### 3.7. Adverse Effect

Data for severe adverse reaction (grade III/IV/V) were extracted from seven trials. 15 types of severe toxicity in patients treated with gefitinib or gefitinib plus chemoradiotherapy were assessed. The pooled RRs of all toxicities, diarrhea, and skin toxicity both showed significant differences for patients who received gefitinib-based therapy (P=0.0003; P=0.03). Instead, other toxicities revealed no differences (Table 2).

### 3.8. Publication Bias

There was no publication bias among trials for ORR, OS, and PFS, according to Begg's test (ORR: P= 0.368; OS: P= 0.462; PFS: P=1.000) and Egger's test (ORR: P= 0.101; OS: P= 0.318; PFS: P= 0.693) (Figure S1).

## 4. Discussion

This meta-analysis provides pooled estimates of the effectiveness and safety of gefitinib-based therapy in patients with advanced HNSCC. Nevertheless, we failed to find a statistically significant difference between gefitinib-based regimens and traditional treatment for the OS or PFS of patients with advanced or recurrent HNSCC. ORR displays similar tendencies as the survival. According to NCCN Clinical Practice Guidelines for Head and Neck Cancers [1], approximately 30% to 40% of patients who present with early-stage disease (stage I or II) are recommended to take single-modality treatment with surgery or radiation therapy (RT). Combined modality therapy is generally recommended to the approximately 60% of patients with locally or regionally advanced disease at diagnosis. For advanced disease, several randomized trials [19–21] and meta-analyses [22, 23] show remarkably improved OS, disease-free survival, and local control when a concomitant or alternating systemic therapy and radiation regimen are compared with RT alone. However, only 30%~40% patients can survive 5 years; thus, novel treatment strategies are urgently needed.

The differences in the response to the treatment of HNSCC, which had been caused by complex primary sites, may affect the result. Bonner's investigation [8] indicated that locoregional control and median overall survival [HR=0.73, 95%CI (0.56-0.95), and P=0.03] were significantly increased in patients treated with RT and cetuximab compared to RT alone, whereas, in their secondary analyses [24] from this trial including only patients with cancer of the larynx or hypopharynx (*n*=168), researchers found that there is no statistically significant difference between the two groups for laryngeal preservation, laryngectomy-free survival, and median overall survival. Therefore, future study on gefitinib in patients with HNSCC of different primary tumors is needed. What is more, further studies to search for genetic variants or specific biomarkers that can contribute to identification of selected patients to benefit from gefitinib are badly needed.

Health-related quality-of-life (QOL) issues are paramount in HNSCC. These tumors have a direct effect on basic physiologic functions like the ability to chew, swallow, and breathe and the senses such as taste, smell, hearing, even uniquely human characteristics like appearance and voice. For head and neck cancer-specific issues, there are three validated and accepted measures: (1) the Functional Assessment of Cancer Therapy Head and Neck module (FACT-H&N) [25], (2) the University of Washington Quality of Life scale (UW-QOL) [26]; and (3) the European Organization for Research and Treatment of Cancer Quality of Life Questionnaire (EORTC-HN35) [27]. Differences exist in the methods of evaluation of QOL in the two trials, so we did not perform meta-analysis. Even so, QOL improvement has been proved in gefitinib arm. Gefitinib is a small-molecule targeting agent that specifically inhibits EGFR-tyrosine kinase, which is usually administered as a once-daily oral tablet. This study shows that gefitinib did not improve the survival of patients with advanced HNSCC, but the subgroup analyses manifest that gefitinib alone and chemotherapeutics (i.e., methotrexate; methotrexate + fluorouracil) are equivalent in ORR in patients with recurrent HNSCC. Simultaneously, gefitinib arm may improve QOL. Hence, gefitinib may be one of the optimal choices for recurrent patients who are refractory to high-intensity therapy. More researches to evaluate the QOL in HNSCC patients with gefitinib are needed.

In our meta-analysis, we found that no significant differences exist between the two groups for most toxicity data except for diarrhea and skin toxicity. The package inserts of gefitinib and literature show that diarrhea and skin toxicity are common adverse reactions, which are usually reversible and manageable with appropriate interventions [28]. Heterogeneity exists in the pooled RRs of the grade 3 to 4 anemic and hemorrhage toxicity. As only two studies reported the two toxicities, we did not undertake sensitivity analysis.

Based on the current evidence, this meta-analysis has several limitations. First, the study size was limited, and too few trials were included for some subgroup analyses, so that we cannot conduct meta-analysis for each disease stage, such as LA or RM. Moreover, this meta-analysis included several small sample studies. Second, some RCTs were of poor quality; for example, they used unclear allocation concealment. Third, different treatment modalities were adopted among the studies. Fourth, primary sites of the head and neck cancer were various in the included studies, but few studies subdivided these lesions, leading to the failure of analyzing them according to the specific disease sites. Fifth, we did not conduct meta-analysis of QOL, which is an essential factor for efficacy, because of the small number of trials and different evaluation methodologies.

## 5. Conclusions

Disappointingly, benefits from gefitinib of patients with advanced HNSCC are still negative. It cannot prolong the OS and PFS or improve ORR, with the cost of increased odds of skin toxicity and diarrhea. Nevertheless, for recurrent patients, gefitinib is a promising agent, which is equivalent to methotrexate and methotrexate + fluorouracil, and tends to improve QOL.

## Figures and Tables

**Table 1:** The characteristics of the included RCTs.

Study	Region	Type of study	Age	No. of patients	Gender (male/female)	Treatment	Follow-up time	duration of treatment	Outcomes
Stewart, 2009	Multi-centre	Phase III RCT	NR	486	396/90	T1: Gefitinib (250 mg/d) T2: Gefitinib (500 mg/d) C: Methotrexate	2 years	until disease progression	OS, ORR, ADR
Gregoire, 2011	Multi-centre	Phase II RCT	NR	226	198/28	T:RT+Cisplatin+Gefitinib C:RT+Cisplatin+ placebo	2 years	a maximum of 2 years after randomization or until disease progression, unacceptable toxicity, patient withdrawal, study closure, or death	OS, ORR, PFS, ADR
Argiris, 2013	America	Phase III RCT	60.8 61.4	270	190/49	T:Gefitinib+Docetaxel C: Docetaxel+placebo	35 months	until progression	OS, ORR, ADR
Singh, 2013	India	RCT	55 53	60	48/12	T:RT+gefitinib C: RT	20 months	90 days; 7 weeks	ORR, ADR
Bhattacharya, 2014	India	RCT	NR	61	54/7	T:RT+Cisplatin+Gefitinib C: RT+Cisplatin	1 year	7 weeks	ORR, ADR
Kushwaha, 2015	India	RCT	47 46.9 46.95	117	111/6	T: Gefitinib C1: Methotrexate C2:Methotrexate+ fluorouracil	27 months	8.89 months; 6.42 months; 6.5 months	ORR, ADR
Saini, 2016	India	RCT	50 54	67	64/3	T:RT+Cisplatin+Gefitinib C: RT+Cisplatin	42 months; 45 months	9 weeks; 7 weeks	OS, PFS, ORR, ADR

RCTs: randomized controlled trials; NR: no report; T: treatment group; C: control group; OS: overall survival; ORR: overall response rate; PFS: progression free survival; ADR: adverse drug reaction.

**Table 2:** Adverse effects associated with gefitinib.

Adverse effect	No. of studies	Model	RR (95%CI)	*P *value	Heterogeneity (*p*,I^2^)	Conclusion
Diarrhea	6	Fixed	4.29(1.96, 9.41)	0.0003	P=0.62; I^2^=0%	Positive
Mucositis	6	Fixed	1.20 (0.96, 1.50)	0.10	P=0.86, I^2^=0%	Negative
Skin toxicity	6	Fixed	1.71 (1.06, 2.74)	0.03	P=0.24; I^2^=26%	Positive
Dysphagia	4	Fixed	0.90 (0.57, 1.42)	0.65	P=0.17; I^2^=40%	Negative
Nausea	4	Fixed	0.98 (0.47, 2.02)	0.95	P=0.55; I^2^=0%	Negative
Vomiting	4	Fixed	1.01 (0.49, 2.10)	0.97	P=0.60; I^2^=0%	Negative
Weight loss	3	Fixed	0.48 (0.12, 1.92)	0.30	P=0.73; I^2^=0%	Negative
Fibrosis	2	Fixed	0.77 (0.43, 1.40)	0.40	P=1.00; I^2^=0%	Negative
Neutropenia	2	Fixed	0.97 (0.46, 2.07)	0.94	P=0.92; I^2^=0%	Negative
Leukopenia	2	Fixed	1.20 (0.60, 2.39)	0.60	P=0.88; I^2^=0%	Negative
Oedema	2	Fixed	1.00 (0.55, 1.82)	0.99	P=0.96; I^2^=0%	Negative
Xerostomia	2	Fixed	0.94 (0.41, 2.16)	0.88	P=0.60; I^2^=0%	Negative
Fatigue	3	Fixed	0.97 (0.54, 1.72)	0.91	P=0.27; I^2^=24%	Negative
Anemic	2	Random	1.00 (0.04, 22.86)	1.00	P=0.08; I^2^=67%	Negative
Hemorrhage	2	Random	1.30 (0.07, 24.23)	0.86	P=0.11; I^2^=61%	Negative

## Supplementary Materials

Supplementary Materials Figure S1: Begg's funnel plots for overall response rate (ORR) (A), overall survival (OS) (B), and progression-free survival (PFS) (C). Egger's funnel plots for ORR (D), OS (E), and PFS (F).
