# Supplementary figures and images for: Efficacy and Safety of Gefitinib in Patients with Advanced Head and Neck Squamous Cell Carcinoma: A Meta-Analysis of Randomized Controlled Trials
Source: J Oncol. 2019 May 23;2019:6273438. doi: 10.1155/2019/6273438 (PMC6556337; doi:10.1155/2019/6273438)

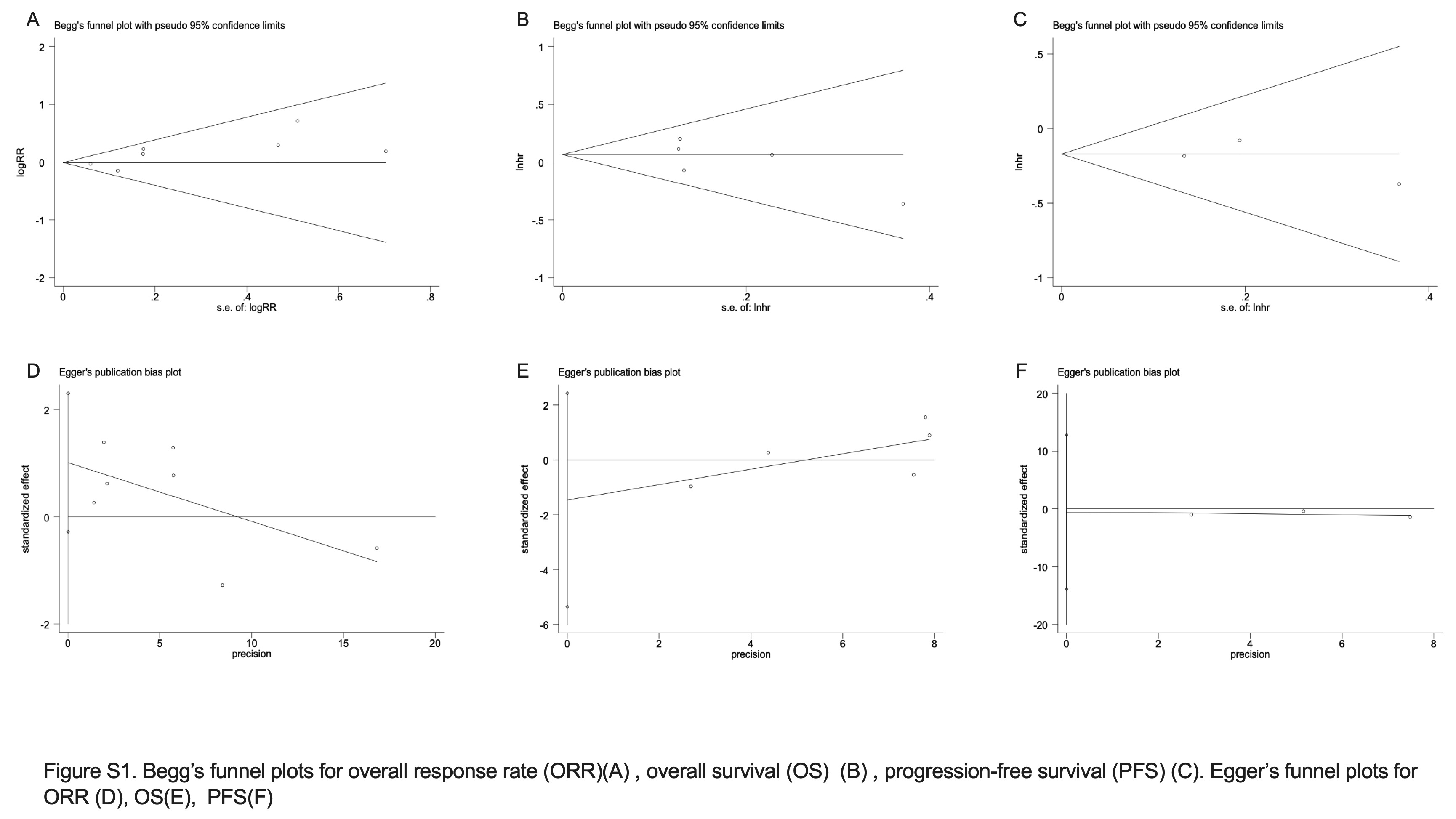

Supplement: Supplementary Materials — Figure S1: Begg's funnel plots for overall response rate (ORR) (A), overall survival (OS) (B), and progression-free survival (PFS) (C). Egger's funnel plots for ORR (D), OS (E), and PFS (F). [file 6273438.f1.jpg]
